# 

**Published:** 1878-09

**Authors:** 


### Chicago Medical Journal

AND

### EXAMINER.

Vol. XXXVII.—SEPTEMBER, 1878.—No. 3.

### To our Readers and Correspondents.

Beginning with Vol. XXXVII., July, 1878, the Chicago Medical Journal and Examiner prints all records of length and weight in terms of the Metric System, and all records of temperature in degrees of the Centigrade Scale. The Metric System, legalized in the United States and Great Britain, is extensively or exclusively employed by other civilized nations, and has thus become an essential part of the international language of science. It is recommended for adoption to the profession in this country by the American Medical Association and other scientific bodies. To-day no physician can afford to be ignorant of its value, its simplicity and the meaning of its terms.

The subjoined tables and scales, which have been kindly prepared for us by Prof. W. S. Haines, will be continuously reproduced in subsequent numbers of this journal, for the ready reference of our readers and correspondents.

15

### Metric Measures of Length.

| | | | |
|---|---|---|---|
| Millimeter | 0.001 | of a Meter | 0.03937 inches. |
| Centimeter | 0.01 | " " | 0.39370 " |
| Decimeter | 0.1 | " " | 3.93707 " |
| **Meter** | **1.** | **Meter** | **39.37079** " |
| Decameter | 10. | Meters | 393.70790 " |
| Hectometer | 100. | " | 3937.07900 " |
| Kilometer | 1000. | " | 39370.79000 " |

### Metrical Weights.

| | | | |
|---|---|---|---|
| Milligram | 0.001 | of a Gram | 0.015 grains. |
| Centigram | 0.01 | " " | 0.154 " |
| Decigram | 0.1 | " " | 1.543 " |
| **Gram** | **1.** | **Gram** | **15.432** " |
| Decagram | 10. | Grams | 154.323 " |
| Hectogram | 100. | " | 1543.234 " |
| Kilogram | 1000. | " | 15434.348 " |

☞ The United States "nickel" five cent piece weighs five grams, and is two centimeters in diameter.

### Approximate Equivalent of Metrical Weights.

*For Rapid Reference.*

| *Milligrams.* | *Grains.* | *Decigrams.* | *Grains.* |
|---|---|---|---|
| 1 (written 0.001 or \|001)* | $\frac{1}{65}$ | 1 (written 0.1 or \|1) | $1\frac{1}{2}$ |
| 2 | $\frac{1}{32}$ | 2 | 3 |
| 3 | $\frac{1}{22}$ | 3 | $4\frac{1}{2}$ |
| 4 | $\frac{1}{16}$ | 4 | 6 |
| 5 | $\frac{1}{13}$ | 5 | $7\frac{1}{2}$ |
| 6 | $\frac{1}{11}$ | 6 | 9 |
| 7 | $\frac{1}{9}$ | 7 | 11 |
| 8 | $\frac{1}{8}$ | 8 | $12\frac{1}{2}$ |
| 9 | $\frac{1}{7}$ | 9 | 14 |

| *Centigrams.* | *Grains.* | *Grams.* | *Grains.* |
|---|---|---|---|
| 1 (written 0.01 or \|01) | $\frac{1}{6}$ | 1 (written 1. or 1\|) | 15 |
| 2 | $\frac{1}{3}$ | 2 | 30 |
| 3 | $\frac{6}{13}$ | 3 | 46 |
| 4 | $\frac{7}{11}$ | 4 | 61 |
| 5 | $\frac{3}{4}$ | 5 | 77 |
| 6 | $\frac{9}{10}$ | 6 | 92 |
| 7 | 1 | 7 | 108 |
| 8 | $1\frac{1}{4}$ | 8 | 123 |
| 9 | $1\frac{1}{3}$ | 9 | 139 |

A Kilogram=$2\frac{1}{5}$ lbs. Avoirdupois.

* The decimal *line* instead of points makes errors impossible.

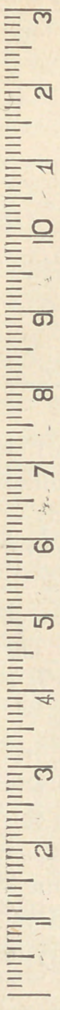

CENTIMETERS AND MILLIMETERS.

### Metric Fluid Measures.

When using the metric system, fluids are preferably prescribed by weight, employing the gram, its multiples and subdivisions, just the same as with solids, thus avoiding the errors due to refraction, adhesion, and inaccurate measuring vessels. For practical purposes four grams of water may be regarded as equivalent to a fluid drachm of that liquid, and the same may be considered true of tinctures and infusions; syrups, on the average, are about one-third heavier than water, so that a fluid ounce of a syrup will be approximately represented by 43 grams.

If preferred, however, fluids may be prescribed by volume, in the metric, just as in the present system, using for that purpose the *Cubic Centimeter*, that is, a volume represented by a cube all of whose sides measure one centimeter. An ordinary back-gammon die is usually about this size. One cubic centimeter (written 1 C. C.) = 16.231 minims. It is approximately regarded as one fourth of a fluid drachm.

### Approximate Equivalents of Cubic Centimeter.

| | | | | |
|---|---|---|---|---|
| 0.001 | C. C. | = ......... | 1/65 | minim. |
| 0.01 | " | = ......... | 1/6 | " |
| 0.1 | " | = ......... | 1 1/2 | " |
| **1.** | **C. C.** | **= ..................** | **15** | **minim.** |
| 4. | " | = ......... | 1 | fluid drachm. |
| 16. | " | = ......... | 4 | fluid drachms. |
| 32. | " | = ......... | 1 | ounce. |

1000 C. C. (usually known as a **Liter**) is a trifle more than one quart, wine measure.

The following prescription—

R: Potassii bromidi, ℥i.

Elixir aurantii, fl., ℥viij.

M.

Would, in metric terms, be written:

Potassic bromide, 32

Orange elixir, 250

### BOOKS AND PAMPHLETS RECEIVED.

Urological Dictionary, Containing an Explanation of Numerous Technical Terms; the Qualitative and Quantitative Methods Employed in Urinary Investigation: the Chemical Characters and Microscopical Appearances of the Normal and Abnormal Elements of the Urine and their Clinical Indications. By J. King, M. D., with twenty-seven useful tables and thirty-nine wood-cuts; pp. 226; leather. Cincinnati: Wilstach, Baldwin & Co.; 1878. Chicago: Jansen, McClurg & Co.

On the Therapeutical Forces: An Effort to Consider the Action of Medicine in the Light of the Modern Doctrine of the Conservation of Force By Thômas J. Mays, M. D., member of the Luzerne County Medical Society, etc., etc. Cloth; pp. 143. Price, $1.20. Philadelphia: Lindsay & Blackiston; 1878. Chicago: Jansen, McClurg & Co.

Anatomy, Descriptive and Surgical: By Henry Gray, F. R. S., etc., etc. With 522 engravings on wood. The drawings by H. V. Carter, M. D., and D. Westmacott. The Dissections jointly by the author and D. Carter. With an Introduction on General Anatomy and Development. By T. Holmes, M. A., Cantab., Surgeon to St. George's Hospital, etc., etc. A new American, from the eighth and enlarged English edition. To which is added Landmarks, Medical and Surgical, by Luther Holden, F. R. C. S., etc. Leather; pp. 983. Price, $7.50. Philadelphia: Lindsay & Blackiston; Chicago: Jansen, McClurg & Co.

Transactions of the Medical and Chirurgical Faculty of the State of Mary land; 80th Annual Session, held at Baltimore, Md., April, 1878.

The Soft Palate; Its Value in Diagnosis as Compared with the Tongue in Derangements of the Liver, Malarial Diseases and Exanthematous Diseases. By William Abram Love, M. D., Professor of Physiology in the Atlantic Medical College, etc., etc. Reprint from the Transactions of the Medical Association of Georgia, 1878.

Medico-Legal Evidence, Relating to the Detection of Human Blood, Presenting the Alterations Characteristic of Malarial Fever, on the Clothing of a Man Accused of the Murder of Narcisse Avrieux, December 27 1876.

Cholocystotomy for the Removal of Gall Stones in Dropsy of the Gall Bladder. By J. Marion Sims, M. D. Reprint from *British Medical Journal*, June 8, 1878.

Report on the Health of Children in the Oneida Community. By T. R Noyes, M. D., Oneida, New York.

Minutes of the State Medical Society of Arkansas, at its Third Annual Session. Little Rock, Arkansas.

Post Nasal Catarrh. By W. Porter, M. D., St. Louis, Mo. Reprint from Illinois *Medical Recorder*, Vandalia, Ill.

Tubercular Laryngitis: By W. Porter, M. D., St. Louis, Mo. Reprinted from the Transactions of the Missouri State Medical Association, 1878.

Fibrous Tumors of the Uterus: By W. H. Byford, M. D., Professor of Obstetrics and Diseases of Women and Children in the Chicago Medical College. One of a Series of American Clinical Lectures, edited by E. C. Seguin, M. D. New York: G. P. Putnam & Sons, 182 Fifth av.

Second Annual Report of the State Board of Health of the State of Wisconsin, for the year ending December 31, 1877.

Transactions of the Iowa State Medical Society, 1877-8, Vol. III.

### ANNOUNCEMENTS FOR THE MONTH.

### SOCIETY MEETINGS.

Chicago Medical Society—Mondays, Sept. 2 and 16.
West Chicago Medical Society—Mondays, Sept. 9 and 23.

### CLINICS.

MONDAY.

Eye and Ear Infirmary—2 to 3 p. m., Ophthalmological, by Prof. Holmes; 3 to 4 p. m., Otological, by Prof. Jones.
Mercy Hospital—2 to 3 p. m., Surgical, by Prof. Andrews.
Rush Medical College—2 p. m., Dermatological and Venereal, by Dr. Hyde. 3 p. m., Medical, by Dr. Bridge.

TUESDAY.

Mercy Hospital—2 p. m., Medical, by Prof. Hollister.

WEDNESDAY.

Mercy Hospital—2 p. m., Eye and Ear, by Prof. Jones.
Rush Medical College—4 p. m., Diseases of the Chest, by Dr. E. Fletcher Ingals.

THURSDAY.

Mercy Hospital—2 p. m., Medical, by Prof. Davis.
Rush Medical College—1:30 p. m., Neurological, by Prof. Lyman.
Eye and Ear Infirmary—2 to 3 p. m., Ophthalmological, by Dr. Hotz.

FRIDAY.

Mercy Hospital—2 p. m., Medical, by Prof. Davis.

SATURDAY.

Rush Medical College—2 p. m., Surgical, Prof. Gunn.
Chicago Medical College—2 p. m., Surgical, by Profs. Andrews and Isham; 3 p. m., Diseases of the Chest, by Prof. Johnson.

Special Clinics daily, from 2 to 4 p. m., at the South Side Dispensary, and at the Central Free Dispensary.

